# High throughput profile-profile based fold recognition for the entire human proteome

**DOI:** 10.1186/1471-2105-7-288

**Published:** 2006-06-07

**Authors:** Liam J McGuffin, Richard T Smith, Kevin Bryson, Søren-Aksel Sørensen, David T Jones

**Affiliations:** 1 The BioCentre, University of Reading, Whiteknights, PO Box 221, Reading RG6 6AS, UK; 2 Department of Computer Science, University College London, Malet Place, London WC1E 6BT, UK

## Abstract

**Background:**

In order to maintain the most comprehensive structural annotation databases we must carry out regular updates for each proteome using the latest profile-profile fold recognition methods. The ability to carry out these updates on demand is necessary to keep pace with the regular updates of sequence and structure databases. Providing the highest quality structural models requires the most intensive profile-profile fold recognition methods running with the very latest available sequence databases and fold libraries. However, running these methods on such a regular basis for every sequenced proteome requires large amounts of processing power.

In this paper we describe and benchmark the JYDE (Job Yield Distribution Environment) system, which is a meta-scheduler designed to work above cluster schedulers, such as Sun Grid Engine (SGE) or Condor. We demonstrate the ability of JYDE to distribute the load of genomic-scale fold recognition across multiple independent Grid domains. We use the most recent profile-profile version of our mGenTHREADER software in order to annotate the latest version of the Human proteome against the latest sequence and structure databases in as short a time as possible.

**Results:**

We show that our JYDE system is able to scale to large numbers of intensive fold recognition jobs running across several independent computer clusters. Using our JYDE system we have been able to annotate 99.9% of the protein sequences within the Human proteome in less than 24 hours, by harnessing over 500 CPUs from 3 independent Grid domains.

**Conclusion:**

This study clearly demonstrates the feasibility of carrying out on demand high quality structural annotations for the proteomes of major eukaryotic organisms. Specifically, we have shown that it is now possible to provide complete regular updates of profile-profile based fold recognition models for entire eukaryotic proteomes, through the use of Grid middleware such as JYDE.

## Background

Maintaining databases that contain the most up-to-date, high quality models of the protein structures for entire proteomes is becoming increasingly difficult. Structural annotation databases such as 3D-Genomics[1], Gene3D[2], Superfamily[3] and the Genomic Threading Database[4,5] contain predicted models for all of the proteins encoded within key genomes. The quality of the models deposited in these databases relies on obtaining the closest templates for each target sequence and constructing the best possible sequence to structure alignment. Most of the methods used to construct models currently rely on sequence-profile based searches; however keeping these models up-to-date is problematic for several reasons. The proteome sequences of key organisms are often updated on a monthly basis, the protein structures within the Protein Data Bank [6] are updated every week and many of the non redundant protein sequence databases [7], used to construct PSI-BLAST [8] profiles for example, are updated every single day. It is clear from the LiveBench experiments [9] that the reliability of fold recognition methods is greatly affected by the templates that are available in a fold library. A hard target sequence, regarded as having an analogous or novel fold one day, may have a homologous template available the next. It is also clear that the quality of sequence profiles is likely to increase with the availability of closer homologues.

Currently the best fold recognition methods are those which employ profile-profile based searching. A number of studies have shown that these methods greatly outperform the sequence-profile based methods which are often used to populate structural annotation databases (see Ohlsen *et al*. for a recent review) [10]. Clearly the most comprehensive structural annotation for a given proteome would be achieved through more rigorous profile-profile based searches. However, the trade-off for the increased coverage of high confidence annotations is the speed at which predictions can be made. The main added computational overhead is due to the required construction of profiles for every unique target sequence within a proteome.

In this paper we describe our JYDE (Job Yield Distribution Environment) system, a meta scheduler designed to be run above cluster schedulers such as Sun Grid Engine [11] and Condor [12] [see Additional File 1]. We demonstrate that JYDE is able to distribute large numbers of intensive fold recognition jobs on demand running across several computer clusters within independent Grid domains. We use the most recent profile-profile version of our mGenTHREADER software [13] in order to annotate the latest ENSEMBL [14] version of the Human proteome as quickly as possible. Using our JYDE system to harness over 500 CPUs from 3 independent Grid domains we have been able to annotate 99.9% of the protein sequences within the human proteome in less than 24 hours. This study demonstrates that the prospect of carrying out on demand snapshots of the structural annotations for key eukaryotic organisms is now entirely feasible.

## Implementation

### Profile-profile based fold recognition using mGenTHREADER

The most recent mGenTHREADER protocol [13] was followed for profile-profile based fold recognition. The comparison method used was designed to directly compare PSI-BLAST position-specific scoring matrix (PSSM) scores, and makes use of an optimized heuristic comparison metric. The target PSSM was built using 10 iterations of PSI-BLAST (-j 10), searching the UNIREF100 sequence data bank with low complexity regions, coiled coil regions and transmembrane segments filtered out. The profile-profile scoring scheme we use is essentially based on the dot product of two PSSM vectors *X *(from the target) and *Y *(from the template), though where any negative values in the target PSSM are set to zero:

S(X,Y)=∑i=120max⁡{0Xi}Yi∑i=120max⁡{0Xi}
 MathType@MTEF@5@5@+=feaafiart1ev1aaatCvAUfKttLearuWrP9MDH5MBPbIqV92AaeXatLxBI9gBaebbnrfifHhDYfgasaacH8akY=wiFfYdH8Gipec8Eeeu0xXdbba9frFj0=OqFfea0dXdd9vqai=hGuQ8kuc9pgc9s8qqaq=dirpe0xb9q8qiLsFr0=vr0=vr0dc8meaabaqaciaacaGaaeqabaqabeGadaaakeaacqWGtbWucqGGOaakcqWGybawcqGGSaalcqWGzbqwcqGGPaqkcqGH9aqpdaWcaaqaamaaqahabaGagiyBa0MaeiyyaeMaeiiEaG3aaiWaaeaafaqabeGabaaabaGaeGimaadabaGaemiwaG1aaSbaaSqaaiabdMgaPbqabaaaaaGccaGL7bGaayzFaaGaemywaK1aaSbaaSqaaiabdMgaPbqabaaabaGaemyAaKMaeyypa0JaeGymaedabaGaeGOmaiJaeGimaadaniabggHiLdaakeaadaaeWbqaaiGbc2gaTjabcggaHjabcIha4naacmaabaqbaeqabiqaaaqaaiabicdaWaqaaiabdIfaynaaBaaaleaacqWGPbqAaeqaaaaaaOGaay5Eaiaaw2haaaWcbaGaemyAaKMaeyypa0JaeGymaedabaGaeGOmaiJaeGimaadaniabggHiLdaaaaaa@59D6@

To find the correct alignment parameters (i.e. gap penalties) for this scoring function, the parameters were optimized using a grid search to maximize the sum of model quality for each top hit across a benchmark set of 50 difficult fold recognition targets. Using a standard affine gap penalty term, a gap-opening penalty of 7.4 and extension penalty of 0.47 were found to be optimal.

A non-redundant template fold library for mGenTHREADER was constructed from 6331 representatives from the PDB. All pairs of proteins within the fold library had low sequence homology to one another (FASTA [15] E > 0.001 and < 30% identity). The profile-profile version of mGenTHREADER was run on the Human proteome sequences downloaded from the ENSEMBL website [14] (Version NCBI35 from November 2005) using the JYDE pipeline described below. The method was also run on the sequences from the fold library itself in order to estimate the reliability of the output scores (see below).

### Sequence-profile based fold recognition using GenTHREADER

The GenTHREADER protocol [16] was used for sequence-profile based fold recognition. The same procedure was carried out as for mGenTHREADER above using the identical fold library and target sequences.

### Calculation of P-values for GenTHREADER and mGenTHREADER fold assignments

It is essential to provide a quantitative measure of the confidence we have in any particular fold assignment from both methods. For this we used an approach similar to that used in a previous study [5] based on hypothesis testing. We determined the statistical significance of obtaining a fold match with a given score or better when compared to a null model. Our null model is based on the criteria that a match of this score or better has occurred by chance and does not actually signify that the sequence has the specified fold. Clearly the alternative model is that the match score is due to the sequence actually having the given fold.

In more detail, we generated random pairings of sequences which are known not to have the same fold. This was carried out by comparing the TM-score of pairs of proteins within the fold library, using the TM-align method and recommended parameters [17]. Applying GenTHREADER and mGenTHREADER to these sequences provided a score distribution for the null model. Unlike our previous study [5], which assumed an extreme value distribution for the null distribution, in this study we fitted a generic density model based on a Gaussian kernel using the R software [18]. This was found to more accurately reflect particular features of our current data. We were then able to determine the statistical significance of any score using a one-sided test based on this distribution. The p-value gives the proportion of non-matching folds which, on average, would be incorrectly assigned as matches. The coverage of sequences with assigned structures was determined within the Human proteomes using standard p-value cut-offs (< 0.001, < 0.01 and < 0.05).

### The JYDE pipeline for high throughput structural annotation

The JYDE (Job Yield Distribution Environment) software package was developed as part of the e-Protein project (e-Protein.org) in order to distribute structural annotation jobs across multiple co-operating processing clusters at independent Grid domains. JYDE consists of a meta-scheduler that works above cluster schedulers, such as SGE or Condor, and supports multiple submission front-ends. The software currently in use at UCL has a Bioinformatics tailored web interface which is powered by a Tomcat servlet. The servlet allows authenticated users to upload a proteome sequence file via a web form and then prepares the data for processing by bioinformatics software such as mGenTHREADER. The proteome sequence files are subdivided into smaller files which are then passed through to the Portal. The Portal maintains the job queues with different priorities for different users and projects.

The Portal requests permission for job execution from the Distribution Manager (DM). The Distribution Manager on the submission server is part of a peer-to-peer network with the DMs at other Grid sites and attempts to balance the load across them. The DM issues permits to the Portal which instructs the portal to execute a particular job at a particular site. The Portal has different modules to support communication with different kinds of clusters, e.g. a specific pluggable module talks to sites which are running SGE6 or Condor. This module submits the jobs to the cluster, reports on their status and returns the results when they finish.

**Figure 1:**
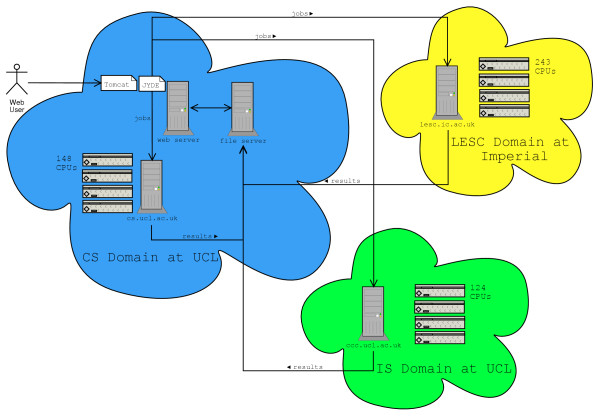
**JYDE pipeline**. Systems involved in the experiment and flow of data – see methods section for a detailed description.

Figure 1 illustrates the flow of data through the JYDE pipeline and gives an overview of all of the components involved in the experiment. The hardware and software components and data flow are described in detail below.

### JYDE hardware setup

Three clusters were used within three independent Grid domains at UCL and Imperial. At the peak of the experiment 148 Pentium IIIs (1.3 GHz) were available at the UCL Computer Science domain (cs.ucl.ac.uk), 243 Opterons (various speeds) were available at the Imperial LESC domain (lesc.imperial.ac.uk) and 192 Xeons (2.8 GHz) were available at the UCL Information Systems domain (ccc.ucl.ac.uk). In total 515 CPUs were available to carry out the structural annotations.

We arranged to have increased access to each of the clusters between the 2nd and 4th December 2005. Exclusive access to the cluster at the UCL Computer Science domain was obtained throughout the experiment. We had increased priority to the cluster at the Imperial LESC domain and restrictions were imposed on the job lengths of other users. At the UCL Information Systems domain restrictions were put in place to prevent any other users from submitting jobs during the experiment.

### JYDE software setup

Three components of JYDE were installed on the submission web server including: the Bioinformatics front end to the Portal (Tomcat servlet), the Portal itself and the Distribution Manager.

Installing our software on the clusters was relatively straightforward and merely required a standard user account to be setup on the front end machine. A tar file containing the binaries for our mGenTHREADER software was uploaded into the NFS-shared home directory on each cluster. Java was also installed in the home directory where it was found to be unavailable. Public keys were setup in order to enable the submission web server to SSH into each clusters and submit jobs. This SSH connection also enabled the clusters to transfer results files back to the main data store on the file server. Finally we configured the mGenTHREADER shell scripts for each cluster. These scripts were run by SGE on each node, which in turn called the mGenTHREADER binaries. The SGE 'qsub' command was run on the front end machine with the shell script and input parameters.

The mGenTHREADER program required read-access to large database files containing up-to-date sequence and structure data. An rsync through SSH was setup to keep these files updated on each cluster.

### Data flow during rapid annotation of the human proteome

We downloaded the latest available human proteome sequence file from ENSEMBL (version NCBI35, November 2005) and submitted it for annotation using mGenTHREADER via the web interface.

The human proteome sequence file contained 32010 unique sequences. For reasons of efficiency, we chose to allocate 5 sequences to each job so that JYDE would run a total of 6402 jobs. The parameters were passed to the Portal, the input file was pre-processed and subdivided into 6402 smaller files.

The Portal queried the Distribution Manager (DM) for permits to run 6402 jobs. The DM initially issued a few hundred permits to the Portal in order to fill each cluster with jobs. Each permit specified the identity of a cluster where the Portal was allowed to run a job. For each of the 6402 waiting jobs, the Portal matched the job up to a permit and then submitted a permit to a specified cluster. For each job the input data file, containing 5 protein sequences, was then uploaded to the cluster via SSH. Since the clusters in this experiment were all running SGE, the job was then submitted using the 'qsub' command via an SSH connection to the cluster front end.

As each of the jobs ran, log files were written containing information about the job status. The log files were then transferred back to the central data store on the file server. The status of each run could then be queried using the web interface on the web server. The Tomcat servlet was able to read the log files and display which jobs were queued, running or completed on each of the clusters.

The DM worked by constantly checking the lengths of the wait queues at each site. When a queue on a particular site fell below a certain threshold, new permits were issued for that site, the Portal would then submit more jobs to that site. The aim of this strategy was to keep every CPU at every site running jobs and to keep a few jobs waiting at each site at any time, but not so many that it would hinder the DM's ability to make scheduling decisions. Prior to running, the majority of jobs were queued in Portal's queue on submission web server and not in the SGE cluster queues on the Grid sites.

## Results

### Throughput of human proteome annotation

**Figure 2:**
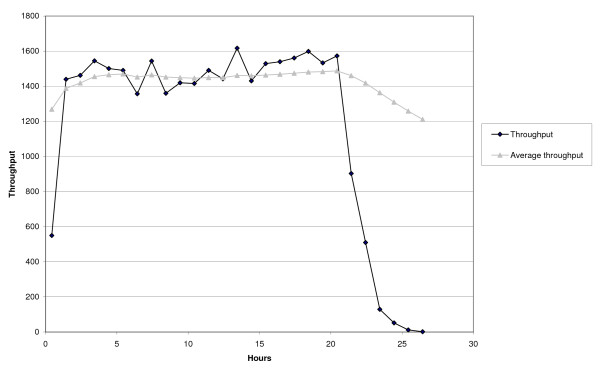
**Throughput of Human proteome annotation**. Throughput per hour measured both as the number of protein sequences annotated per hourly interval (black) and as the cumulative number of sequences completed divided by the number of hours passed (grey).

Figure 2 shows the throughput achieved, measured by both the number of sequences annotated per hourly interval and by the average number of sequences completed per hour. For the main duration of the experiment we maintained a high throughput peaking at an average of 1487 sequences annotated per hour. A maximum throughput of 1617 sequences was achieved during one hourly interval. The plot also shows the expected initial lag phase during the first hourly interval, whilst sequence files were being prepared and transferred to remote sites and whilst jobs were being assigned to nodes. We can also observe the expected "tailing off" phase, where throughput decreases rapidly as the number of nodes available begins to exceed the number of annotation jobs that are left to run.

**Figure 3:**
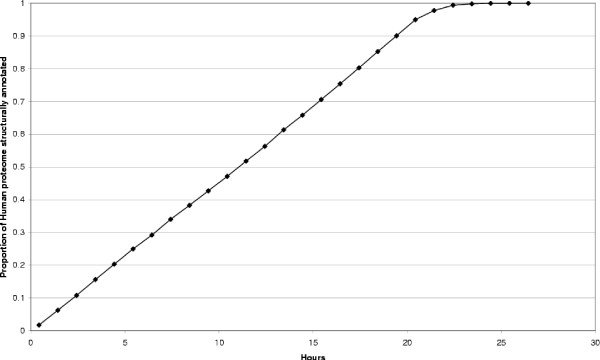
**Throughput of Human proteome annotation**. Throughput measured as the proportion of the human proteome annotated per hour.

The cumulative throughput per hour is shown in Figure 3, where the proportion of annotated sequences in the human proteome is plotted against the hour. The plot remains linear for over 20 hours, which again indicates the constant rate of throughput that was achieved. Approximately 99.9% of sequences within the human proteome were structurally annotated in under 24 hours. The expected tailing off phase takes the overall time to just over 26 hours for completion of every single sequence.

### CPU usage at independent Grid domains

**Figure 4:**
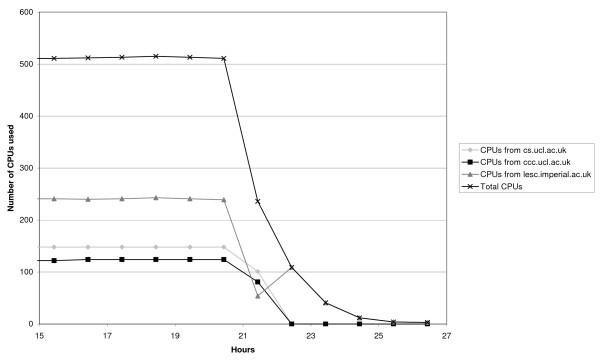
**CPU usage**. Number of CPUs used from each cluster at each independent Grid domain (cs.ucl.ac.uk, ccc.ucl.ac.uk and lesc.imperial.ac.uk), calculated as the number of individual jobs running at each cluster per hourly interval. For the majority of the experiment the total number of CPUs remained over 500. The tail end of the experiment is shown where the number of CPUs available begins to exceed the number of sequences left to process.

Figure 4 shows the number of CPUs used per hourly interval, for the last 10 hours of the experiment. Excluding the tailing-off phase, an average of 504 CPUs were used during the experiment and at its peak 515 CPUs were available.

The tailing-off phase in CPU usage is clearly indicated on the plot (Figure 4). This occurs when the number of CPUs exceeds the number of jobs left to run. Towards the end of the run the last remaining jobs were redirected to the fastest cluster (lesc.imperial.ac.uk) in attempt to minimize the tailing-off effect.

### Comparison of GenTHREADER and mGenTHREADER: estimated coverage of the human proteome

**Figure 5:**
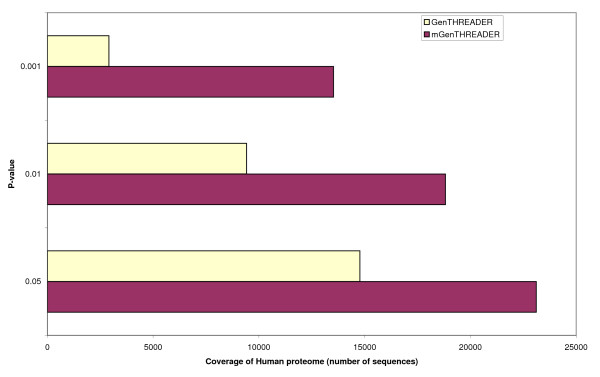
**Estimated coverage of Human proteome**. Coverage of sequences with assigned structures at standard p-value cut-offs (< 0.001, < 0.01 and < 0.05). The sequence-profile method GenTHREADER is compared to the more rigorous profile-profile version of mGenTHREADER. See methods section for explanation of p-values.

Figure 5 shows the difference in estimated sequence coverage between the sequence-profile method GenTHREADER [16,19] and the profile-profile version of mGenTHREADER [13]. Clearly the profile-profile version of mGenTHREADER outperforms GenTHREADER in terms of sequence coverage at each level of confidence (see Implementation section for calculation of p-values). Approximately 46% of the sequences with globular regions from the latest version of the Human proteome (14755/32010) can be confidently assigned folds (p < 0.05) using GenTHREADER, compared to over 72% (23112/32010) using the latest mGenTHREADER method. Over 10000 more sequences (approximately 1/3 of the Human proteome) can be annotated at the highest confidence level (p < 0.001) using mGenTHREADER than GenTHREADER.

### Comparison of GenTHREADER and mGenTHREADER: processing benchmarks

Table 1 shows the difference in processing requirements between GenTHREADER and mGenTHREADER running on 148 identical nodes from the cs.ucl.ac.uk domain (1.3GHz Pentium IIIs). The sequence-profile method GenTHREADER is approximately 5.4 times faster on average than the profile-profile version of mGenTHREADER. On a single PIII 1.3GHz processor it would take less than half a year to carry out GenTHREADER predictions for the entire Human proteome, yet it would take about 2.4 years to carry out the equivalent mGenTHREADER predictions.

### Analysis of latest available templates

The PDB [6] is updated on a weekly basis. During the week that the fold library was constructed (2^nd ^November, 2005), 15 new structures became available which had no detectable sequence homology to known templates within the fold library. These structures have the following PDB codes and chain identifiers: 2esnA, 2es7A, 2bryA, 2beiA, 2bduA, 2bdqA, 2axwA, 2auaA, 2aneA, 2ahuA, 1z7aA, 1ytlA, 1ys4A, 1ylqA, 1on1A, 1wp7A.

These latest template structures were assigned to 211 protein sequences within the Human proteome using mGenTHREADER. Of these 211 sequences, 23 had new assignments which were estimated to be significant (p < 0.05).

**Figure 6:**
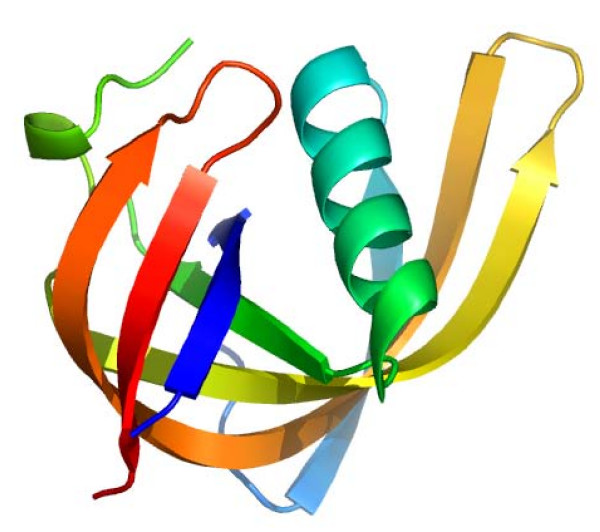
**A confident model of a previously unannotated domain built from a newly available template**. Model built from novel template with the PDB code 2ane region 292–393 of the human protein sequence ENSG00000170500 with p < 0.001. At the time of writing this structure has no assigned SCOP [20] or CATH [21] code and only has a close DALI alignment to one other structure (1zbo). Prior to the week that the fold library was created for the experiment, no structural assignment could have been made to this region with that level of confidence. The template (2ane), along with 14 others with low sequence homology to known folds, was deposited into the PDB during the week that the fold library was created. During the experiment these new templates were assigned to 211 protein sequences from *Homo sapiens*, 23 of which were found to be significant (p < 0.05).

Figure 6 shows an example model built from novel template with the PDB code 2aneA, an ATP-dependant protease. At the time of writing this protein has no detectable sequence homology to any other known structure and has not been assigned a SCOP [20] or CATH [21] code. The protein only has one other structural relative (1zbo), which was identified using the DALI server [22]. The mGenTHREADER method assigned the template 2aneA to residues 292–393 of the human protein sequence ENSG00000170500 with p < 0.001. Prior to the week that the fold library was created for the experiment, no structural assignment could have been made to this region with that level of confidence. According to a recent ENSEMBL search, a number of GO terms (GO:0006510 – ATP-dependent proteolysis and GO:0004176 – ATP-dependent peptidase activity) have been mapped to this entry via UniProt/RefSeq, which agree with this structural assignment.

## Discussion

In this study we have provided a proof of concept for carrying out on demand profile-profile fold recognition for large eukaryotic proteomes. We have developed and benchmarked Grid middleware in the form of a meta-scheduler called JYDE [see Additional File 1]. This first version of JYDE has been designed specifically for distributing fold recognition software such as mGenTHREADER for large-scale structural annotations but it is also easily extensible to other bioinformatics applications.

Profile-profile based methods for fold recognition are able to detect more remote homologues with higher confidence than can be found with sequence-profile based methods [10]. Figure 5 clearly demonstrates the advantage of using the profile-profile version of mGenTHREADER for annotation of the Human proteome over the sequence-profile method GenTHREADER. There have been many studies on the advantages of profile-profile methods over sequence-profile methods since the first method developed by Rychlewski and colleagues [23]. In a recent review, Ohlson *et al*. [10] observed that profile-profile methods performed at least 30% better than standard sequence-profile methods both in alignment quality and in their ability to recognize distantly related proteins. This observation is reflected in the difference in estimated coverage of significant fold assignments obtained by mGenTHREADER over GenTHREADER (Figure 5).

It is important that structural annotation of whole proteomes can be updated on demand in order to ensure that the most accurate models are obtained for every sequence. New fold templates are released on a weekly basis and new sequences appear daily. The example model in Figure 6 was constructed from one of 15 new templates which were only made available during the week that the fold library was constructed. This fold was assigned to a domain of a human protein where no structural template was previously available. So why not just align sequences to the new structures incrementally, as and when they come out? On the surface this may appear to be a pragmatic approach; however the sequence databases from which profiles are constructed are also updated frequently. This means that the ranking of models may change as more accurate sequence to structure alignments can be made. This would, in turn, mean that model rankings from week-to-week could not be accurately compared against one another. Additionally, versions of proteome sequences are often updated on a monthly basis which would further complicate updates, were they to be carried out incrementally. Finally, other structure prediction applications which make use of sequence profiles would also benefit from continual updates. For example Przybylski *et al*. [24] observed that secondary structure prediction improves as sequence databases increase in size. Secondary structure alignment scores using predicted secondary structures have been an important feature of many fold recognition methods [16]. It is clear from the LiveBench [9] assessments that the highest quality fold recognition models are produced by profile-profile based methods which maintain both continually updated fold libraries and sequence databases.

The trade off for increased accuracy is the speed at which predictions can be made. The profile-profile methods such as mGenTHREADER are significantly more CPU intensive than the sequence-profile methods such as GenTHREADER (Table 1). However, through the development of JYDE we have provided a Grid platform to enable proteome wide profile-profile fold recognition to be carried out on demand. The system could be easily scaled up to include more clusters and is extensible to other high throughput CPU intensive bioinformatics applications.

While it may not be necessarily economic to carry out a complete update of the structures for just the Human proteome every 24 hours, it is perhaps necessary to carry out an update at least every month in order to maintain accurate models for the latest sequence versions. This could be carried out in line with the new release cycle of the proteome sequence. If we were to use the JYDE system in its current setup to run mGenTHREADER predictions 24 hours a day, it would be possible (and perhaps more justifiable) to carry out complete structural annotations for up to 31 or so large eukaryotic proteomes per month, in line with their ENSEMBL release versions.

The JYDE system is not restricted to protein fold recognition and could be applied to any bioinformatics applications where there is a need to regularly distribute intensive methods on large-scale data sets. The system could also be deployed in situations where the performance of a stand-alone prediction server would have insufficient power, for example serving *ab initio*/new fold predictions for individual sequences on demand.

Although the current version of the JYDE system has proved to be robust and efficient there are some aspects which could be further improved. For instance, we are aware that the current method initialises a large number of SSH sessions, and the Distribution Manager is not yet optimally configured for job allocation. We are hoping to address these issues in the next version of the software.

It is clear that the tailing off phase could be further reduced in order to decrease the total time taken. One option would be to reduce the number of sequences per job to one at the end of the run, as the number of sequences left to run equals the total number of CPUs available. It is impractical to initially set the number of sequences per job to one. This is because of the inefficiencies of handling 32010 jobs in the initial submission, which hinders performance at the start of the run. However, it should be possible to build in dynamically configurable job sizes in future versions of JYDE.

Another consideration for reducing the tailing off phase would be to redirect all final jobs to the fastest cluster when the number of sequences left to run equals the number of nodes available in the fastest cluster. This was attempted at the end of the second run where we redirected the last remaining jobs to the Imperial-LESC cluster (lesc.imperial.ac.uk) (Figure 4). It is difficult to accurately predict how long a fold recognition job will need to run based on the sequence information alone. However, it would be possible to monitor long running jobs and resubmit them to faster nodes in the early stages of the run. Each of these performance improvements can be carried out automatically and will be added to future versions of JYDE, indeed it is our priority to minimize the tailing off phase as far as possible.

## Conclusion

We succeeded in annotating 99.9% of the Human proteome in under 24 hours. The JYDE system was able to effectively schedule jobs dynamically across three different Grid domains, achieving a maximum throughput of 1487 sequences per hour and using 515 CPUs at the peak of the run. This study clearly demonstrates the feasibility of on demand high-throughput structural annotations of the proteomes of major eukaryotic organisms. The use of grid middleware such as JYDE software should allow us to maintain continually updated structural annotation databases containing the highest possible quality models of protein structures for key eukaryotic organisms.

## Availability and requirements

• **Project names: **e-Protein, JYDE

• **Project home pages: **, 

• **Operating system(s): **Platform independent (Linux/UNIX preferred)

• **Programming language: **Java 1.5

• **Other requirements: **Ant

• **License: **Freely available for academic use

• **Any restrictions to use by non-academics: **License required

## Authors' contributions

LJM and RTS conceived, designed and carried out the experiment. LJM drafted the paper and developed the structural annotation pipeline and web portal. RTS contributed text to the manuscript, developed the distribution manager and maintained the hardware. KB performed the statistical analysis. SS conceived of the JYDE system and participated in the design and coordination of the study. DTJ developed and contributed the key protein structure prediction software and carried out final editing of the manuscript. All authors have read and approved the final manuscript.

## Supplementary Material

Additional File 1 JYDE software. Job Yield Distribution Environment software, see README file for installation instructions. Click here for file

## Figures and Tables

**Table 1:** Processing benchmarks for GenTHREADER versus mGenTHREADER. The processing time required for the sequence-profile method GenTHREADER is compared to the profile-profile version of mGenTHREADER. The data was collected by running both methods on sequences from the Human proteome using 148 identical CPUs (1.3 GHz Pentium IIIs from the cs.ucl.ac.uk domain) over a period of 10 hours. The throughput was measured as the mean number of sequences with structural annotations per hour.

	GenTHREADER	mGenTHREADER
Mean sequences per hour on 148 identical CPUs	1236	230
Mean sequences per hour on 1 CPU	8.35	1.55
Mean time for 1 Human sequence on 1 CPU (min)	7.18	38.61
Estimated time for all Human sequences on 1 CPU (days)	159.70	858.24
