# Supplementary material for: High throughput profile-profile based fold recognition for the entire human proteome
Source: BMC Bioinformatics. 2006 Jun 7;7:288. doi: 10.1186/1471-2105-7-288 (PMC1513610; doi:10.1186/1471-2105-7-288)
Supplement: Additional File 1 — JYDE software. Job Yield Distribution Environment software, see README file for installation instructions. [file 1471-2105-7-288-S1.bz2 › jportal2/build/hello.jsp]

Sample Application JSP Page


|  |  |
| --- | --- |
|  | Sample Application JSP PageThis is the output of a JSP page that is part of the Hello, World application. It displays several useful values from the request we are currently processing. |

|  |  |
| --- | --- |
| Context Path: | <%= request.getContextPath() %> |
| Path Information: | <%= request.getPathInfo() %> |
| Query String: | <%= request.getQueryString() %> |
| Request Method: | <%= request.getMethod() %> |
| Servlet Path: | <%= request.getServletPath() %> |
