# Supplementary material for: High throughput profile-profile based fold recognition for the entire human proteome
Source: BMC Bioinformatics. 2006 Jun 7;7:288. doi: 10.1186/1471-2105-7-288 (PMC1513610; doi:10.1186/1471-2105-7-288)
Supplement: Additional File 1 — JYDE software. Job Yield Distribution Environment software, see README file for installation instructions. [file 1471-2105-7-288-S1.bz2 › jportal2/build/index.html]

Distributed mGenTHREADER


|  |  |
| --- | --- |
|  | |
|  | |
|  |
| GTD  home> | **JPortal - Distributed proteome annotation for the GTD** |
|  |
|  | |
| Info | This form allows you to submit a proteome sequence for annotation using a selected program. The annotation job will be distributed across clusters at University College and Imperial College. Further information and references can be found on the Genomic Threading Database home page . |
|  | |
| Select   Proteome | |  | | --- | | **File to upload:** | | **Organism name:** | |
|  | |
| Select   Program | |  | | --- | | **PSIBLAST and PSIPRED** | | **PSIPRED** | | **GenTHREADER** | | **mGenTHREADER** | | **nFOLD** | | **Number of proteins per node:** | |  | |
|  | |
| Filtering   Options | |  | | --- | | Turn **ON**  pfilt | | - Turn **OFF** transmembrane masking | | - Turn **OFF** coiled-coil masking | | - Turn **ON** compositional masking | | - Turn **OFF** low-complexity masking | | |
|  | |
| Submit | |  | | --- | |  | |
|  | |
| contact |
| ---   McGuffin, L. J., Street, S., Bryson K., Sorensen, S. A. & Jones, D. T. (2004) The Genomic Threading Database: a comprehensive resource for structural annotations of the genomes from key organisms. Nucleic Acids Res., 32, D196-D199.   --- | |
| UCL home | Bioinformatics home | GTD home | McGuffin home | | | | |
