# Supplementary material for: High throughput profile-profile based fold recognition for the entire human proteome
Source: BMC Bioinformatics. 2006 Jun 7;7:288. doi: 10.1186/1471-2105-7-288 (PMC1513610; doi:10.1186/1471-2105-7-288)
Supplement: Additional File 1 — JYDE software. Job Yield Distribution Environment software, see README file for installation instructions. [file 1471-2105-7-288-S1.bz2 › jportal2/build/status.jsp]

<%@page import="receiver.\*"%>
<%@page import="common.\*"%>
<% Config c = new Config(); int id=new Integer(request.getParameter("id")); %>
<% MyRpcClient xmlrpc = new MyRpcClient (c.getXmlRpcUrl()); %>
Job <%= id %> status: <%= xmlrpc.execute("getStatus",id) %>
Results
  
  
Reload this page.
