# Supplementary material for: High throughput profile-profile based fold recognition for the entire human proteome
Source: BMC Bioinformatics. 2006 Jun 7;7:288. doi: 10.1186/1471-2105-7-288 (PMC1513610; doi:10.1186/1471-2105-7-288)
Supplement: Additional File 1 — JYDE software. Job Yield Distribution Environment software, see README file for installation instructions. [file 1471-2105-7-288-S1.bz2 › jportal2/README.pdf]

README

Introduction

This is a preview release of JYDE2. The original JYDE prototype was a proof-of-concept for the JYDE architecture and was highly specialised for running certain Bioinformatics jobs on our own clusters. JYDE2 is a clean, modular rewrite, intended to make it possible to extend and maintain the code in future, and to be installed on systems other than our own. It can currently be used to run simple jobs, and we have already implemented a new queing system that would not have been possible on JYDE 1. However, many of the features essential to a real production system have yet to be implemented. (See TODO for a list.)

Authors

Richard Smith (r.smith@cs.ucl.ac.uk) Liam McGuffin (l.mcguffin@cs.ucl.ac.uk) Soren-Aksel Sorensen (S.Sorensen@cs.ucl.ac.uk)

Requirements

- Java 1.5
- SGE 6 with DRMAA libraries installed. Not all binary distributions of SGE include the DRMAA libraries so you may have to compile your own from source. (If you don't have SGE you may only run in 'TestCluster' mode)
- If you want to use the web front end you will require a servlet container. We tested with Apache Tomcat 5.
- Apache Ant 1.6.5
- For testing purposes, instead of the web front end we use the Xmlrpc-c 1.03 command line tools.

News

Jan 2006 - New queing system implemented. Test script test.sh will submit a bunch of jobs to test it.

Support

This preview release is not intended for production use and there is no support.

TODO

- Write more automated JUnit tests for queing system. [low priority]
- Write database backend for JobStore (use Hibernate?). No need to put JobQueue into database since it can be rebuilt from JobStore at any time. [medium priority]
- Put user accounts in database and implement authentication (plaintext password would be easy, XML-RPC built-in authentication would be better) [high priority]
- Test with latest version of SGE6 on real cluster. [low priority but very easy]
- Write a Receiver and make necessary modifications to support some real-world jobs. [high priority]
- Implement Grid Distribution Manager. This could be based on Alan's GriDM implementation, or it could be copied from the fairly simple hack we did for the human genome run. [medium priority]
- Load config from (XML?) files. This will mean simply rewriting Config.java to parse XML files rather than use hard coded values - it should not require changes anywhere else. [low priority but very easy to do]
- Currently JPortal has to run on a cluster submission host so it can do its qsubs. We need to write proxy classes so the JPortal sends jobs to a local 'ProxyCluster' class which then transparently sends them via RPC to another machine which is running, for example, DrmaaCluster class. This enables us to run JPortal whereever we like and have multiple clusters. (Alternatively, DrmaaCluster could be rewritten to use SSH to connect to remote hosts itself, but if we later added CondorCluster we would need to rewrite the SSH code, so using a proxy class is nicer.) [high priority]

Installation

Download sources from CVS. For historical reasons this requires you to have an account in the 'nrs' usergroup. Someone should get around to migrating to a SVN repository owned by a 'jyde' group eventually:

```
export CVS_RSH=ssh
export CVS-
ROOT=:ext:YOUR_USERNAME@amy.cs.ucl.ac.uk:/cs/research/nets/common1/marine/grs/cvs
cvs checkout jportal2
```

Then compile:

```
cd jportal2
ant
```

If you've downloaded a binary distribution then the previous steps are obviously not necessary.
All necessary libraries are included.
To run the unit tests (optional):

```
ant test
```

Configuration

Currently you have to edit source files and recompile. src/portal/Config.java contains the scheduling interval (seconds), the port to run XML-RPC on, and the URL of the GridM to use.

Also change 'new DrmaaCluster' to 'new TestCluster' if you dont have a SGE6 cluster setup for testing.

We also have two queing systems that are selectable here, SimpleQueue and SorenQueue.

To change the logging level edit data/log4j.properties.

Running

Before you can run the portal you need to have a GridM running:

```
ant gridm
```

Leave GriDM running and open a new shell. If you are using SGE and you have an SGE env setup script, run it now, e.g.:

```
/opt/sge6/default/common/settings.sh
```

Note that currently the portal must run on the cluster's front-end node. You will also need SGE compiled with DRMAA and you will need to edit build.xml to tell Java where to find the DRMAA libraries. This is the section you need to edit:

```
<sysproperty key="java.library.path"
             value="/Users/richard/sge6/lib/darwin/"
             />
```

Then run the portal:

```
ant portal
```

Receivers

To use the portal you need a receiver. If you don't have one, you can make your own, i.e. send XML-RPC calls directly to the portal using the xmlrpc command line tools, which you can download from http://xmlrpc-c.sourceforge.net/

Submit a job, (shell command echo), with input data "blah", owned by "richard" with taskid 1 (taskid is a way of grouping jobs together into tasks, doesnt do much yet.):

```
./xmlrpc-c-1.03/tools/xmlrpc/xmlrpc http://localhost:9548 sub-
mitJob "echo hello" "blah" "richard" i/1
Result: 0
```

The result you get back is your job's id number. You can use to query the status:

```
./xmlrpc-c-1.03/tools/xmlrpc/xmlrpc http://localhost:9548 getStatus i/0
Result: job finished
```

And to return the job's output:

```
./xmlrpc-c-1.03/tools/xmlrpc/xmlrpc http://localhost:9548 getResults i/0
Result: hello
```

There is a script, test.sh, which submits several simple jobs.

Web receiver

A simple web receiver is under development. You will need Tomcat running to use it.(e.g. run /opt/tomcat5/bin/startup.sh) Edit build.xml and enter your Tomcat admin password, then:

```
ant install
```

Or if it is already installed:

```
ant reload
```

Point your webbrowser at http://localhost:8080/bioinf/simple.html

There seems to be a bug in Tomcat on some platforms (e.g. Linux) that prevents it from working with Java 1.5 JSPs. To fix this bug, see here: http://forum.java.sun.com/thread.jspa?threadID=579806&messageID=2928513

Workflow

- Receiver sends HTML form to webbrowser (simple.html)
- User fills in form, browser sends it back to Receiver (servlet: Receiver.java)
- Receiver submits job via XML-RPC request ("submitJob [command] [data] [owner] [taskid]") to Portal (RPC-server: JPortal.java).
- Portal adds job to queue and submits permit request via XML-RPC ("requestPermit [URL_to_send_permit_to]") to GridM (RPC-server: GridM.java).
- GridM submits permit via XML-RPC ("addPermit [name_of_cluster]") to Portal (RPC-server: Portal.java).
- Portal's Scheduler thread wakes up, sees there is a job in the queue and a permit is available, pops them both, and submits the job to the cluster named in the permit. An object of type Cluster which represents the cluster is pulled out of ClusterFactory. In this case the type is DrmaaCluster which knows how to submit jobs to SGE6 clusters.
- Portal's DrmaaCluster submits the job to SGE and records the SGE job ID number.
